# Cytochrome C catalyzed oxygen tolerant atom-transfer radical polymerization

**DOI:** 10.1186/s40643-022-00531-5

**Published:** 2022-04-04

**Authors:** Peng-Cheng Xie, Xue-Qing Guo, Fu-Qiao Yang, Nuo Xu, Yuan-Yuan Chen, Xing-Qiang Wang, Hongcheng Wang, Yang-Chun Yong

**Affiliations:** 1 Biofuels Institute, School of Environment and Safety Engineering, Jiangsu University, 301 Xuefu Road, Zhenjiang, 212013 China; 2 Joint Institute of Jiangsu University-Hongrun Tech, Jiangsu University, 301 Xuefu Road, Zhenjiang, 212013 China

**Keywords:** Atom-transfer radical polymerization, Cytochrome, ATRPase, Biocompatibility, Aerobic condition

## Abstract

Atom-transfer radical polymerization (ATRP) is a well-known technique for controlled polymer synthesis. However, the ATRP usually employed toxic heavy metal ionas as the catalyst and was susceptible to molecular oxygen, which made it should be conducted under strictly anoxic condition. Conducting ATRP under ambient and biocompatible conditions is the major challenge. In this study, cytochrome C was explored as an efficient biocatalyst for ATRP under biocompatible conditions. The cytochrome C catalyzed ATRP showed a relatively low polymer dispersity index of 1.19. More interestingly, the cytochrome C catalyzed ATRP showed superior oxygen resistance as it could be performed under aerobic conditions with high dissolved oxygen level. Further analysis suggested that the Fe(II) embed in the cytochrome C might serve as the catalytic center and methyl radical was responsible for the ATRP catalysis. This work explored new biocompatible catalyst for aerobic ATRP, which might open new dimension for practical ATRP and application of cytochrome C protein.

**Graphical Abstract:**

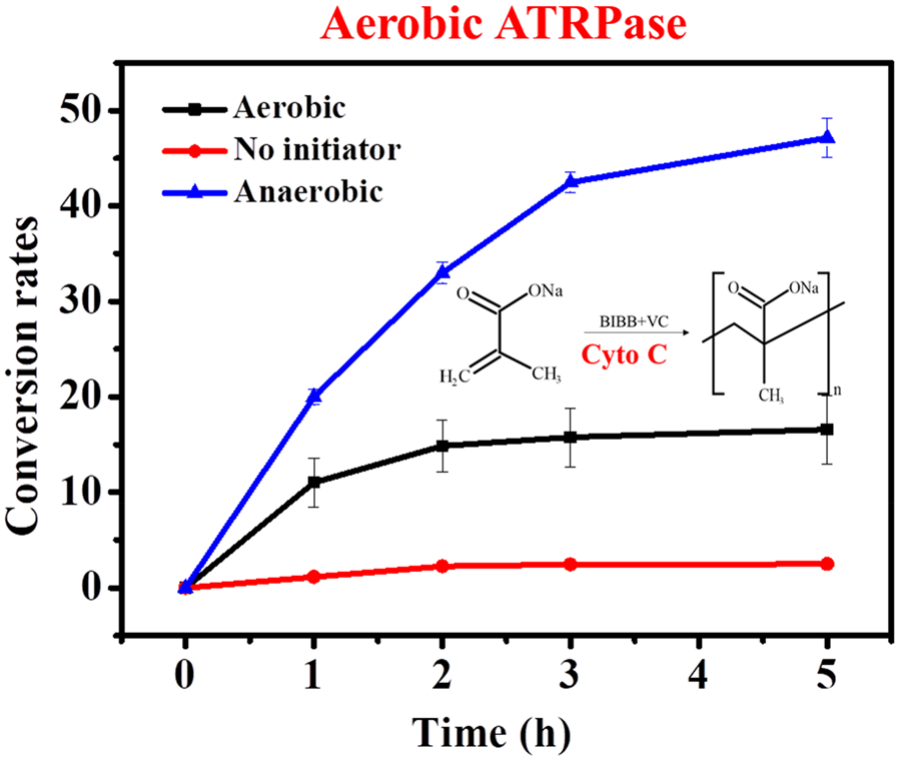

**Supplementary Information:**

The online version contains supplementary material available at 10.1186/s40643-022-00531-5.

## Introduction

Polymer was considered as one of the most widely used materials in the modern society. Since the landmark work in the middle of 1990s, controlled radical polymerization have attracted much attention both in the academic research and practical applications (Pan et al. 2018; Wang and Matyjaszewski 1995). In particularly, atom-transfer radical polymerization (ATRP) became one of the most prevalent polymerization techniques owing to its excellent capability to synthesis of polymer with well-defined topology, precise molecular weight and uniform chain length (di Lena and Matyjaszewski 2010). In a typical ATRP system, the halide initiator reacted with the lower oxidation state transition metal catalyst/activator, which resulted in the generation of organic radical and higher oxidation state metal halide. The organic radical would react with the monomer and enabled chain propagation. Meanwhile, the halogen atom was transferred back from the metal halide to the polymer radical, forming atom transfer equilibrium, which allowed all the polymer chains grew at the same rate to enable well-defined and narrow molecular weight distribution (Ouchi et al. 2009). However, as the heavy metal ions that used as the catalyst was difficult to be removed from the synthesized polymer, serious concern of biotoxicity was raised.

Therefore, efforts have been made to reduce the heavy metals concentration and develop other more biocompatible catalysts. By rational design the Cu complexes, the concentration of Cu(I) used in ATRP was decreased from > 10,000 to < 100 ppm (Ribelli et al. 2018). In another aspect, different transition metals with lower toxicity such as Fe, Co and Ni were explored as alternative catalysts for ATRP catalysts (Ando et al. 1997; Dadashi-Silab and Matyjaszewski 2020; Debuigne et al. 2005). More impressively, it had been found that some proteins/enzymes/cells showed catalytic activity for ATRP, which greatly improved the biocompatibility and made ATRP was more practical to bio-related conditions (Silva et al. 2013; Yuan et al. 2020). The use of bio-component as the catalysts (also termed as ATRPase) provided the possibility to extend the ATRP to various biological related conditions. Therefore, exploration more proteins with ATRP catalytic activity was important and desirable.

Owing to molecular oxygen would quench the radicals and oxidize the catalyst/activators, conventional ATRP required strictly anoxic conditions, which largely limited its applications (Szczepaniak et al. 2021). Thus, various strategies had been invented to in-situ remove the dissolved oxygen, which resulted in the development of oxygen tolerant ATRP (Szczepaniak et al. 2021; Yeow et al. 2018). Inspiring by oxygen-consuming reactions of biological systems, several enzymes including glucose oxidase, horseradish peroxidase were used to in-situ consume the dissolved oxygen and enabled ATRP to tolerant limited amounts of oxygen (Liarou et al. 2018; Matyjaszewski et al. 2017; Pan et al. 2017). These methods usually depended on the enzymatic transformation of O_2_ to CO_2_ or other oxygenated intermediates. These in-situ deoxygenation strategies provided the possibility to conduct ATRP under oxygen-containing conditions, and greatly broadened the applications of ATRP (Szczepaniak et al. 2021). More recently, using the whole cell of *Shewanella oneidensis* MR-1 as the oxygen scavenger, the dissolved oxygen in the ATRP system could be quickly consumed and the ATRP could be conducted under ambient condition (Fan et al. 2020). However, these strategies relied on the consumption of dissolved oxygen to protect the ATRase, which required additional reagents and substrates.

Thus, there are remains a keen interest to discover the ATRPase that could resist oxygen itself as it might largely simplify the aerobic ATRP system. Cytochrome C was ubiquitous redox protein that played important roles in cell respiration. It was also involved in the extracellular electron transfer for whole-cell mediated ATRP (Fan et al. 2018; Nothling et al. 2021). Recently, it was reported that cytochrome C could serve as the catalyst to generate different radicals, which implied this protein might play some roles in radical reactions (Iwahashi et al. 2002; Yu et al. 2011; Yurkova et al. 2009). However, whether the cytochrome C could serve as ATRPase was still unclear. With this context, the ATRP catalytic activity of cytochrome C was determined. The ATRPase activity of cytochrome C protein was verified by HPLC, NMR, and GPC analyses. To further extend the application of the cytochrome C mediated ATRP, its oxygen tolerance was also identified. Moreover, the mechanism for this cytochrome C mediated oxygen tolerant ATRP was proposed.

## Materials and methods

### Chemicals

Sodium methacrylate (SMA), sodium P-styrene sulfonate (PSS), cytochrome C, ascorbic acid, copper (I) bromide (CuBr), 2,2-bipyridyl (bpy), Tris(2pyridylmethyl)amine (TPMA), 1,10-Phenanthrolinemono hydrate, α-Bromoisobutyryl bromide (BIBB), and 5,5-Dimethyl-1-pyrroline-1-oxide (DMPO), were analytical grade, purchased from Sinopharm Group Co. Ltd. (China) and used without further purification.

### ATRP mediated by cytochrome C

Prior to polymerizations, stock solutions of cytochrome C (10× stock from 88.4 mg of cytochrome C per 10 mL of deionized water) and ascorbic acid (10× stock from 1.76 g of ascorbic acid per 100 mL of deionized water) were prepared. Then, a 5 mL polymerization reaction mixture was prepared in a 20 mL bottle as the following, cytochrome C (0.5 mL of stock solution), monomer solution (0.054 g SMA or 0.103 g PSS), ascorbic acid (0.5 mL of stock solution), and 4 mL PBS were mixed. Next, BIBB (initiator, 1.45 µL) was added to initiate the ATRP polymerization. The final concentrations for the reagents were cytochrome C (1 mM), monomer (100 mM), ascorbic acid (10 mM), and initiator (1 mM). For anaerobic ATRP, the dissolved oxygen in the solutions was removed by purging with nitrogen gas for 30 min before polymerization initiation in anaerobic workstation, and the reaction vessel was tightly sealed to maintain the anaerobic conditions. For aerobic ATRP, the oxygen removal step was omitted and the reaction vessel was opened in the air with shaking (180 rpm) during the whole polymerization process.

### Analysis and characterizations

SMA concentration was determined by LC-20 (Shimadzu, Japan) with an HPX-87H column (300 × 7.8 mm, Bio-Rad, USA) and a refractive index detector (the mobile phase is 4 mM H_2_SO_4_ solution, 0.4 mL/min). ^1^H NMR spectra of monomer/polymer solutions were collected on a Bruker Avance III HD 400 MHz NMR spectrometer using D_2_O as solvent (the reaction mixture was centrifuged (12,000 rpm, 8 min) and freeze-drying for 24 h, then the sample was ground into powder and dissolved in D_2_O). Polymer samples were analyzed by gel permeation chromatography (GPC) with Agilent PLgel MIXED-B 10 μm columns (PL1110-6100) using Tetrahydrofuran as the eluent and an 18-angle laser light scattering (MALLS) detector (Silva et al. 2013). Free radical trapping agent of DMPO was added into the ATRP system and was analyzed by electron spin resonance spectroscopy (ESR, Bruker A300, Germany) (Xia et al. 2011).

## Results and discussion

### Discovery of ATRPase activity of cytochrome C

**Fig. 1:**
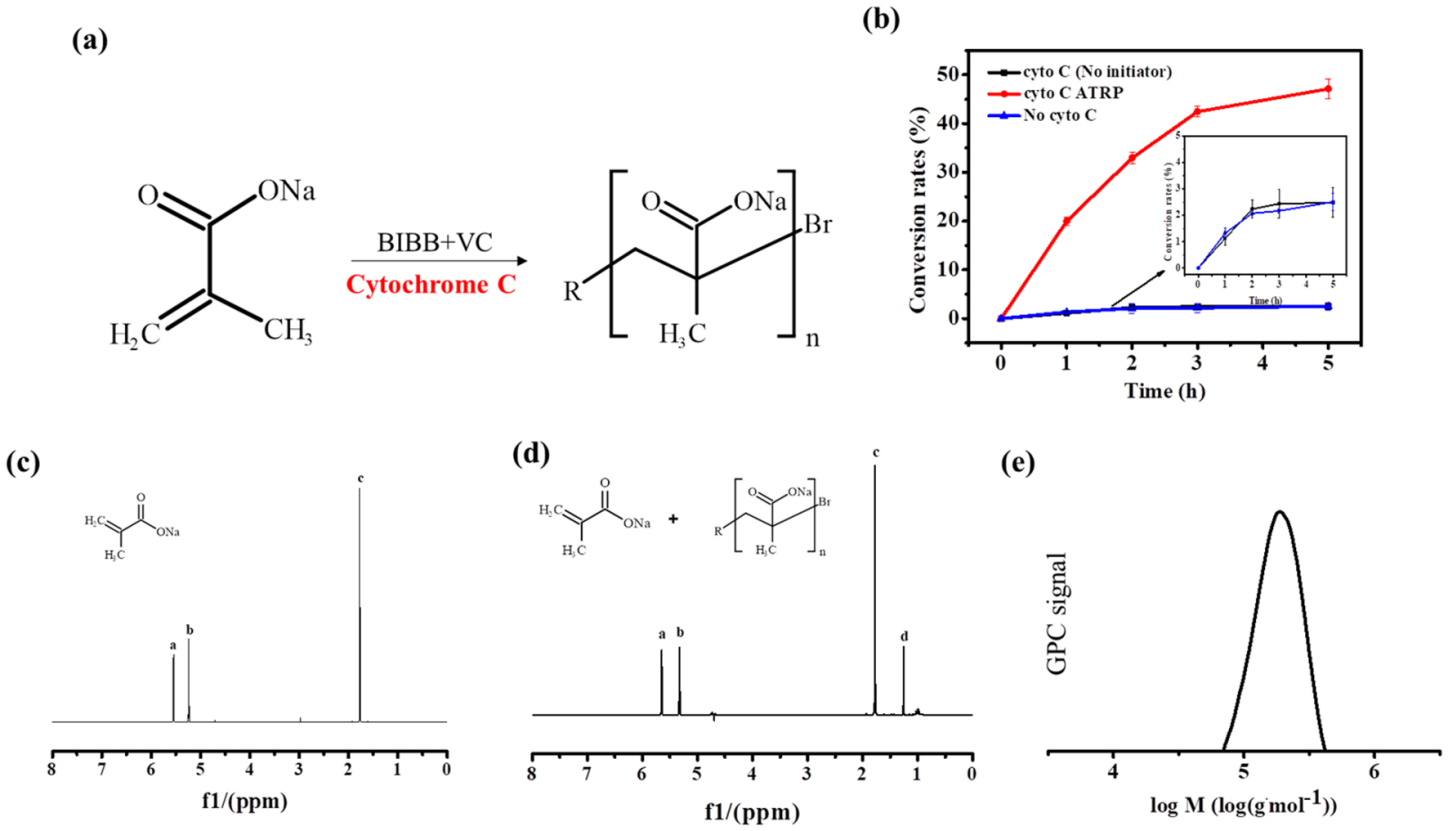
**a** Schematic of cytochrome C catalyzed ATRP polymerization of SMA to PSMA. **b** Conversion of SMA by cytochrome C catalyzed ATRP under anaerobic condition. Inset is the enlarged view of the selected lines. **c** ^1^H NMR spectrum of SMA monomer. **d** ^1^H NMR spectrum of PSMA/SMA mixture synthesized by cytochrome C catalyzed ATRP under anaerobic condition. **e** GPC curve of the PSMA synthesized by cytochrome C catalyzed ATRP under anaerobic condition

To test the possibility of using cytochrome C protein as the catalyst for ATRP polymerization, the bio-ATRP system was developed as the following. SMA was selected as the model molecule for polymerization, while α-bromoisobutyryl bromide (BIBB) was used as the initiator, VC served as the reducing agent. Protein of cytochrome C from bovine was selected as the catalyst (Fig. 1a).

By directly monitoring the monomer conversion of the cytochrome C catalyzed ATRP system, it was found that the SMA monomer was successfully converted (Fig. 1b). After 3 h’ incubation, the conversion rate could reach 40%, while the highest conversion rate of 48.6% was achieved at 5 h. The system without cytochrome C did not show any SMA conversion. In addition, the system contained cytochrome C but without the initiator also showed no conversion, which excluded the monomer adsorption by cytochrome C proteins and also ruled out the possibility that the ATRP was catalyzed by the impurities from glassware or other reagents. As expected, the concentrations of cytochrome C significantly affected the conversion rate, while the optimal concentration obtained was 1 mM (Additional file 1: Fig. S1). Furthermore, the NMR was performed to determine whether the monomer was converted to corresponding polymer or not. For SMA monomer, the ^1^H NMR spectrum showed three main peaks related to the monomer molecule (Fig. 1c). As expected, after polymerization, the ^1^H NMR spectrum exhibited a new peak “d”, suggesting new chemical bound formed (Fig. 1d). Furthermore, the GPC analysis was conducted to determine the molecular weight of the synthesized polymers. The molecular weight of SMA monomer was 108 g mol^−1^. According to the GPC results (Fig. 1e), the average molecular weight (*M*_n_) of the polymer synthesized was 157,900 g mol^−1^. Impressively, the polymer synthesized by the cytochrome C catalyzed ATRP showed a relatively narrow molecular distribution with a polymer dispersity index (PDI) of 1.19, indicating excellent molecular control capability. These results substantiated that cytochrome C could serve as the ATRPase for successfully catalyzing the ATRP polymerization. As cytochrome C proteins are ubiquitous in different organisms and with high biocompatibility, the finding of their ATRPase activity would expand the application of cytochrome C protein and also extend the practical application of ATRP to bio-related conditions.

### Aerobic ATRP catalyzed by cytochrome C

**Fig. 2:**
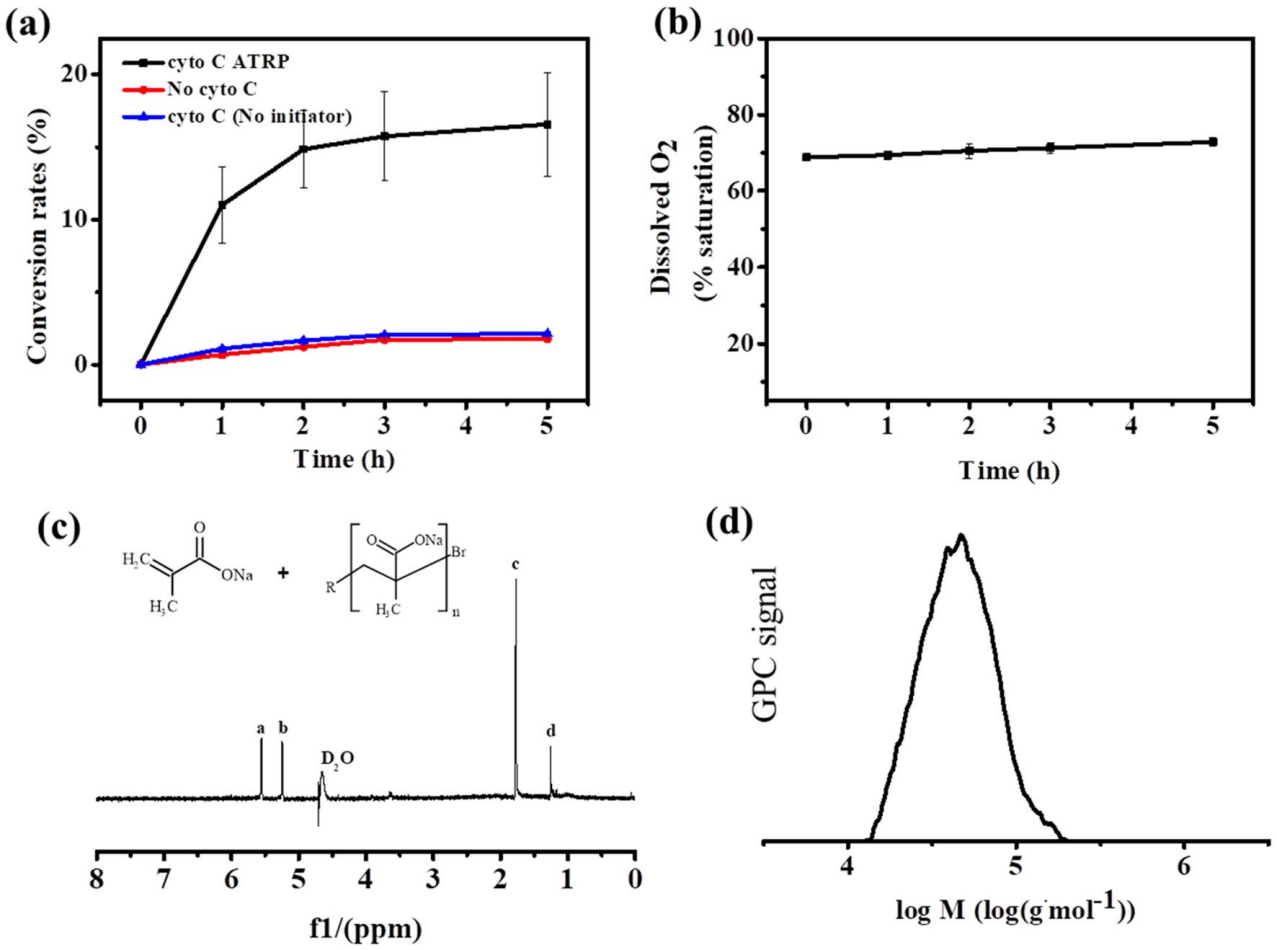
**a** Conversion of SMA by cytochrome C catalyzed ATRP under aerobic condition. **b** Dissolved oxygen (DO) of the aerobic ATRP solution. The DO was presented as the percentage of the oxygen level in solution over the total oxygen level under oxygen saturation condition. **c** ^1^H NMR spectrum of PSMA/SMA mixture synthesized by cytochrome C catalyzed ATRP under aerobic condition. **d** GPC curve of the PSMA synthesized by cytochrome C catalyzed ATRP under aerobic condition

As cytochrome C usually involved in the electron transfer process for redox biological process, such as aerobic respiration (Yu et al. 2020), it was deserved to test whether this protein could catalyze the ATRP process under aerobic conditions or not. Surprisingly, obvious SMA conversion was observed with different cytochrome C concentrations (Additional file 1: Fig. S1) and it was found that over 10% of the SMA monomer was successfully converted with 1 mM cytochrome C (Fig. 2a). After 5 h’ incubation, about 16.6% monomer was converted. It was worth to noted that this ATRP was performed under vigorous shaking in the open air, which enabled the system always contained high dissolved oxygen (the oxygen level in the solution was over 70% of saturation during the whole ATRP process) (Fig. 2b). Then, the reaction products were collected and subjected for NMR analysis. According to the NMR spectrum, there was a new peak of “d” was presented after reaction, which suggesting PSMA was produced (Fig. 2c). Furthermore, GPC was further used to confirm the formation of PSMA and to determine the molecular weight of the polymer. As shown in Fig. 2d, a peak with the *M*_n_ of 38,500 g mol^−1^ was observed, confirming the production of PSMA by the aerobic ATRP reaction. Compared to the anaerobic conditions, the conversion rate under aerobic condition was much lower and the *M*_n_ of the produced PSMA was also smaller. It was reasonable that the molecular oxygen might compete and scavenge the radicals of the ATRP system, which might inhibit the polymerization (Szczepaniak et al. 2021). Nevertheless, the PDI only increased from 1.19 under anaerobic condition to 1.26 under aerobic condition, indicating the robust for molecular weight controlling by cytochrome C mediated ATRP. All the results indicated that cytochrome C protein could catalyze the ATRP under high oxygen level. To the best of our knowledge, this is the first protein based ATRPase that could catalyze the ATRP in the open air. Compared to other ATRP under aerobic conditions (Fan et al. 2020; Szczepaniak et al. 2021), this is the first aerobic ATRP that was conducted under oxygen level higher than 50% of oxygen saturation, which might broad the application area and make the ATRP more practical.

**Fig. 3:**
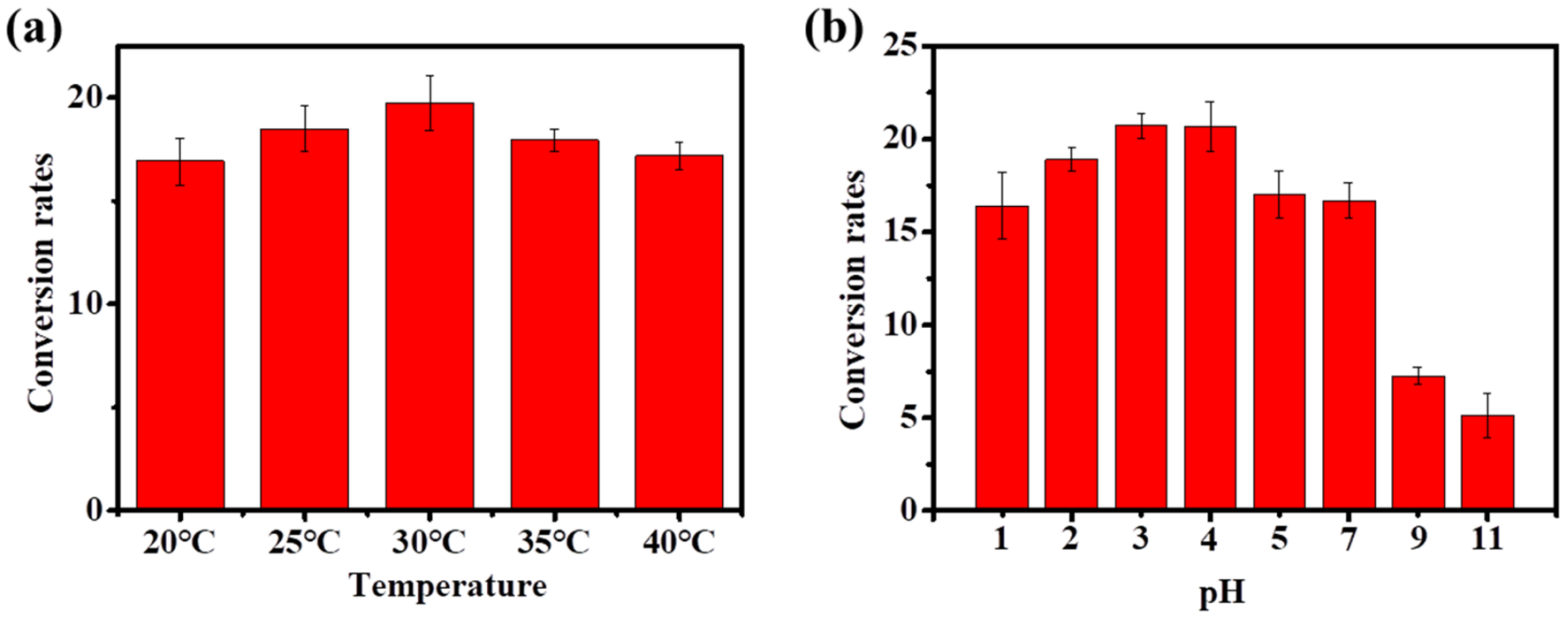
**a** Effect of temperature on the SMA conversion of cytochrome C catalyzed ATRP under aerobic condition. **b** Effect of pH on the SMA conversion of cytochrome C catalyzed ATRP under aerobic condition

Next, the effects of temperature on the cytochrome C catalyzed ATRP were determined. It was found that there was no big difference on the conversion rate within the temperature range of 20 °C to 40 °C (Fig. 3a), suggesting this ATRP could be conducted at broad range of temperature. Since pH is important parameter for enzyme catalysis, the effect of pH on the ATRPase activity of cytochrome C was analyzed (Fig. 3b). At pH > 9, the SMA conversion rate was decreased to lower than 10%, while the conversion rate was higher than 15% at the acidic conditions. The highest conversion rate of about 21% was reached at pH 3.0–4.0. According to the results, the optimum pH for cytochrome C mediated ATRP was about pH 3.0–4.0, which was similar to that of the hemoglobin mediated ATRP (Silva et al. 2013). Moreover, the cytochrome C catalyzed ATRP could also be applied to synthesis other polymer (PSS) both under anaerobic and aerobic conditions (Additional file 1: Figs. S2, S3), suggesting the feasibility of the cytochrome C mediated ATRP for different applications. Compared to other protein mediated ATRP (Table 1), the polymer synthesized by cytochrome C catalyzed ATRP under anaerobic condition showed higher *M*_n_ and relatively low PDI, which showed better performance than most of these obtained by other proteins. More impressively, the PDI obtained by the aerobic ATRP was 1.26, indicating excellent molecular controlling capability. These results suggested that the cytochrome C would be a promising ATRPase for practical application under different conditions, especially was superior to other ATRPases under aerobic conditions.

**Table 1:** Comparison of cytochrome C catalyzed ATRP with recently reported representative protein catalyzed ATRP

Monomer	Catalyst	Reducing agent/additive	Initiator	pH	Time (h)	Conversion (%)	*M*_n_ (g/mol)	PDI (*M*_w_/*M*_n_)	Aerobic/anaerobic	References
PEGMA	LTV	VC	EBiB	7.0	1.0	20.0	2.72 × 10^5^	2.43	Anaerobic	(Ng et al. 2011b)
PEGMA	DhHP-6	Sodium l-ascorbate	EBiB	6.5	2.0	80.7	6.02 × 10^3^	1.08	Anaerobic	(Wang et al. 2018)
PEGMA	DhHP-6@ZIF-8	l-Ascorbate	BPN	8.0	4.0	85.5	8.20 × 10^3^	1.10	Anaerobic	(Jiang et al. 2017)
PEGA	CBL	VC	EBiB	N/A	8.0	50.0	9.81 × 10^3^	1.61	Anaerobic	(Ng et al. 2011a)
NIPAAm	Hematin	Sodium l-ascorbate	EBiB	N/A	24.0	80.0	3.18 × 10^4^	1.80	Anaerobic	(Yamashita et al. 2013)
OEOMA	Mesohemin-(MPEG550)2	Sodium l-ascorbate	PEG2000-Br^a^	7.0	6.0	60.0	6.30 × 10^4^	1.19	Anaerobic	(Simakova et al. 2013)
OEOMA	Hemin	Sodium l-ascorbate	PEG2000-Br^a^	7.0	18.0	50.0	6.00 × 10^4^	1.32	Anaerobic	(Simakova et al. 2013)
OEOMA	HRP	Acetylacetonate	EBPA	6.0	0.5	58.0	3.84 × 10^4^	1.13	Anaerobic	(Matyjaszewski et al. 2018)
BMA	GOx	Sodium pyruvate	EBPA	N/A	6.5	89.0	3.24 × 10^4^	1.80	Anaerobic	(Wang et al. 2018)
NIPAAm	Hb	Sodium nitrate	BIBB	4.0	16.7	N/A	N/A	N/A	Anaerobic	(Divandari et al. 2017)
SMA	Cyto C	VC	BIBB	4.0	5	21.6	3.85 × 10^4^	1.26	Aerobic	This study
SMA	Cyto C	VC	BIBB	7.2	5	48.6	1.58 × 10^5^	1.19	Anaerobic	This study

*PEGMA* poly(ethylene glycol)methyl ether methacrylate, *PEGA* poly(ethylene glycol) methyl ether acrylate, *NIPAAm* *N*-isopropylacrylamide, *OEOMA* oligo(ethylene oxide) methyl ether methacrylate, *BMA* 1 *n*-Butyl methacrylate, *SMA* Sodium methacrylate, *LTV* laccase from *T. versicolor*, *Hb* hemoglobin from bovine blood, *DhHP-6* deuterohemin-β-Ala-His-Thr-Val-Glu-Lys, *ZIF-8* zeolite imidazolate framework-8, *CBL* catalase from bovine liver, *HRP* hemoprotein horseradish peroxidase, *Gox* glucose oxidase from *Aspergillus niger*, *Cyto C* cytochrome C, *VC* vitamin C, *BPN* bromopropionitrile, *EBiB* ethyl a-bromoisobutyrate, *PEG*_*2000*_ polyethylene glycol 2000, *EBPA* ethyl α-bromophenylacetate, *BIBB* α-bromoisobutyryl bromide

### Mechanism of cytochrome C catalyzed ATRP

**Fig. 4:**
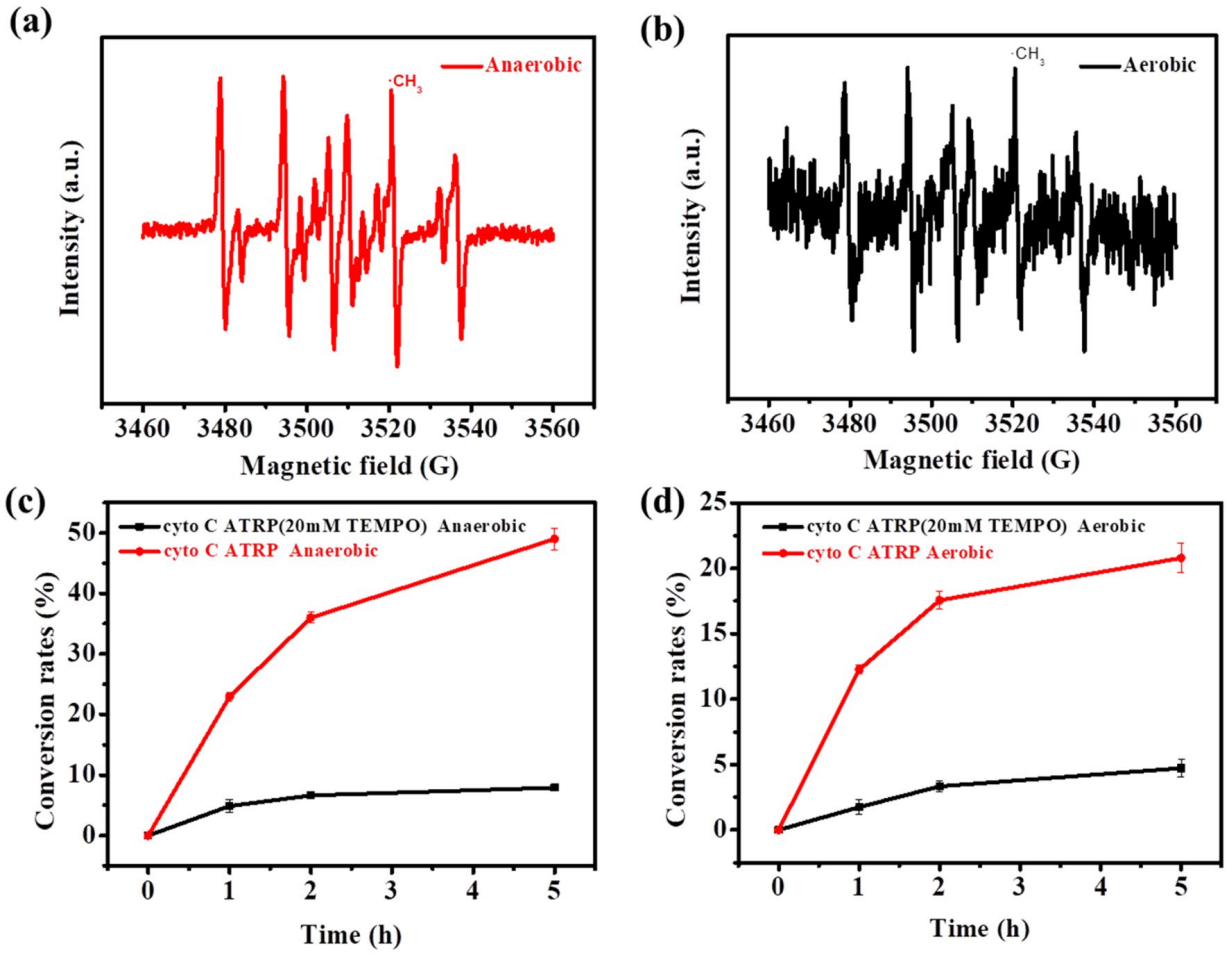
**a** ESR spectrum of cytochrome C catalyzed ATRP solution under anaerobic condition. **b** ESR spectrum of cytochrome C catalyzed ATRP solution under aerobic condition. **c** Effect of radical scavenger (TEMPO) on the SMA conversion of cytochrome C catalyzed ATRP under anaerobic condition. **d** Effect of radical scavenger (TEMPO) on the SMA conversion of cytochrome C catalyzed ATRP under aerobic condition

To get further understanding of the cytochrome C catalyzed ATRP, the radical production during the polymerization was characterized by ESR spin-trapping analysis. Using the DMPO as a spin-trapping agent, the ESR spectroscopic analysis showed obvious DMPO-C radical adduct (Fig. 4a) (Xia et al. 2011). It revealed the generation of methyl radical during the SMA polymerization catalyzed by cytochrome C under the anaerobic condition, indicating the cytochrome C catalyzed ATRP was a typical methyl radical polymerization process. Furthermore, the ESR spectrum of aerobic ATRP catalyzed by cytochrome C was also determined. As shown in Fig. 4b, it was obvious that the spectrum was similar to that from the anaerobic condition, indicating methyl radical was also involved in the cytochrome C catalyzed ATRP under aerobic condition. To further confirm the importance of the radical production in the cytochrome C catalyzed ATRP, the effect of radical scavenger on the SMA conversion was analyzed. As expected, the addition of TEMPO as the radical scavenger significantly suppressed the SMA polymerization for both of aerobic and anaerobic ATRP (Fig. 4c, d), confirming that the cytochrome C catalyzed ATRP performed via the methyl radicals process.

Moreover, it was interesting to further understand which component was responsible for the catalytic activity of cytochrome C. It was reported that iron-based complexes in different enzymes and proteins (horseradish peroxidase, catalase, hemoglobin, etc.) could serve as the catalytic center of ATRPase (Ng et al. 2011a; Rodriguez et al. 2018; Sigg et al. 2011; Simakova et al. 2013). Cytochrome C was a protein-containing iron porphyrin group (Collman et al. 2004; Marques 2007). Thus, the change of Fe(II) was monitored during the ATRP polymerization (Additional file 1: Fig. S4). It was found that the concentration of Fe(II) decreased along with the polymerization. Moreover, the addition of different metal ion chelators (EDTA, DTPA, bpy) dramatically inhibited the polymerization (Additional file 1: Fig. S5). Moreover, both of Fe-TPMA and free Fe(II) could catalyze the anaerobic or aerobic ATRP conversion of SMA (Additional file 1: Fig. S6). Taking together, these results implied that the cytochrome C might rely on the catalysis of iron porphyrin group for ATRP polymerization (Additional file 1: Fig. S7), while the embedding of Fe(II) in the protein might responsible for the oxygen tolerance. However, the detailed mechanism of the oxygen tolerant ATRP deserved further investigation.

In conclusion, a cytochrome C protein catalyzed copper-free ATRP for polymeric materials synthesis was developed. Further analysis explored that this ATRP system was tolerant to high oxygen level. To the best of our knowledge, this is the first ATRP that performed under the aerobic condition with continuously exposure to high dissolve oxygen, which would provide more opportunities for practical ATRP under ambient conditions.

### Supplementary Information

**Additional file 1: Figure S1.** The effect of cytochrome C concentrations on the SMA conversion rate. **Figure S2.** The cytochrome C catalyzed ATRP for the polymerization of PSS. The monomer of PSS do not show fluorescence (Ex: 485 nm; Em: 535 nm), while the polymerized PSS showed obvious fluorescence with the excitation of 485 nm. The results indicated that the PSS monomer could be polymerized by cytochrome C catalyzed ATRP under anaerobic or aerobic condition. **Figure S3.** GPC analysis of PSS polymer synthesized by the cytochrome C catalyzed ATRP. **Figure S4.** The time-course change of the Fe(II) concentration of the cytochrome C catalyzed ATRP under anaerobic condition. **Figure S5.** The effects of different metal ion chelators (2 mM) on the SMA conversion of the cytochrome C catalyzed ATRP under anaerobic condition. **Figure S6.** SMA conversion of the free Fe(II) or Fe-TPMA catalyzed ATRP under anaerobic or aerobic condition. **Figure S7.** The proposed schematic for cytochrome C catalyzed ATRP.

## Data Availability

All data and materials are available in the main text and Additional file.
