# Supplementary material for: Cytochrome C catalyzed oxygen tolerant atom-transfer radical polymerization
Source: Bioresour Bioprocess. 2022 Apr 4;9(1):41. doi: 10.1186/s40643-022-00531-5 (PMC10992558; doi:10.1186/s40643-022-00531-5)
Supplement: Supplementary file 1 — Additional file 1: Figure S1. The effect of cytochrome C concentrations on the SMA conversion rate. Figure S2. The cytochrome C catalyzed ATRP for the polymerization of PSS. The monomer of PSS do not show fluorescence (Ex: 485 nm; Em: 535 nm), while the polymerized PSS showed obvious fluorescence with the excitation of 485 nm. The results indicated that the PSS monomer could be polymerized by cytochrome C catalyzed ATRP under anaerobic or aerobic condition. Figure S3. GPC analysis of PSS polymer synthesized by the cytochrome C catalyzed ATRP. Figure S4. The time-course change of the Fe(II) concentration of the cytochrome C catalyzed ATRP under anaerobic condition. Figure S5. The effects of different metal ion chelators (2 mM) on the SMA conversion of the cytochrome C catalyzed ATRP under anaerobic condition. Figure S6. SMA conversion of the free Fe(II) or Fe-TPMA catalyzed ATRP under anaerobic or aerobic condition. Figure S7. The proposed schematic for cytochrome C catalyzed ATRP. [file 40643_2022_531_MOESM1_ESM.docx]

# *Additional file 1*

# Cytochrome C catalyzed oxygen tolerant atom-transfer radical polymerization

Peng-Cheng Xie^1^, Xue-Qing Guo^2^, Fu-Qiao Yang^1^, Nuo Xu^1^, Yuan-Yuan Chen^1^, Xing-Qiang Wang^1^, Hongcheng Wang^1^, Yang-Chun Yong^1,2,^*

*^1^Biofuels Institute, School of Environment and Safety Engineering, Jiangsu University, 301 Xuefu Road, Zhenjiang 212013, China*

*^2^Joint Institute of Jiangsu University-Hongrun Tech, Jiangsu University, 301 Xuefu Road, Zhenjiang 212013, China*

*Corresponding author, Email: ycyong@ujs.edu.cn


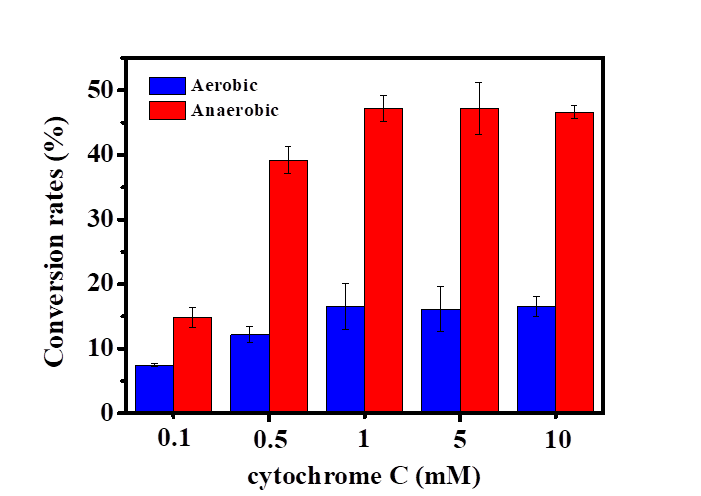


**Fig. S1**. The effect of cytochrome C concentrations on the SMA conversion rate.


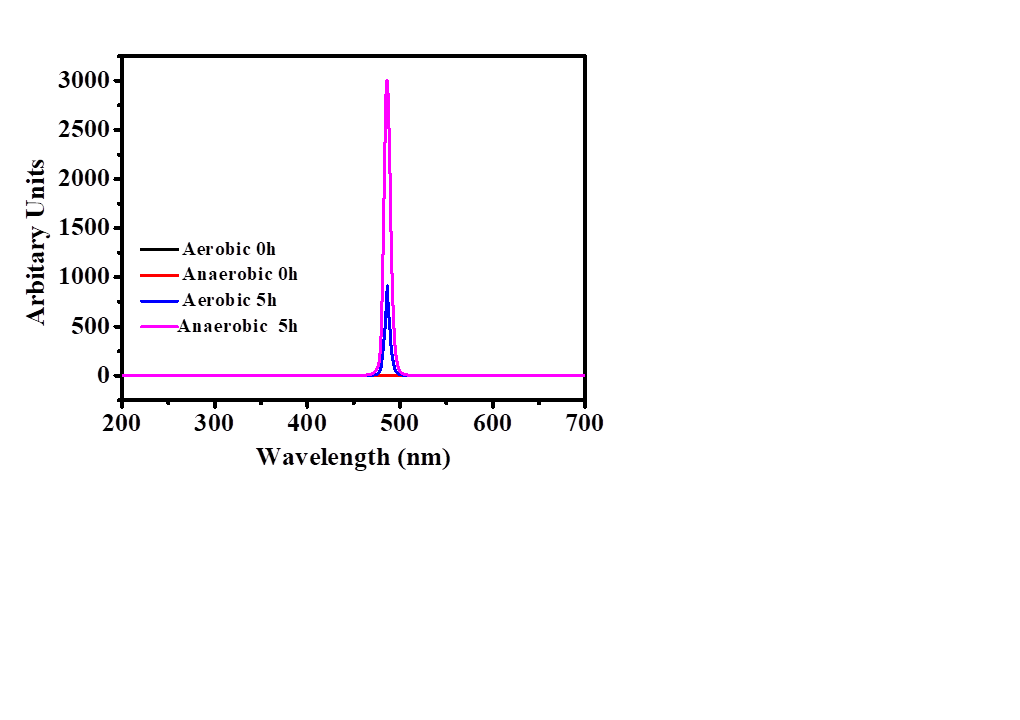


**Fig. S2.** The cytochrome C catalyzed ATRP for the polymerization of PSS. The monomer of PSS do not show fluorescence (Ex: 485 nm; Em: 535 nm), while the polymerized PSS showed obvious fluorescence with the excitation of 485 nm. The results indicated that the PSS monomer could be polymerized by cytochrome C catalyzed ATRP under anaerobic or aerobic condition.


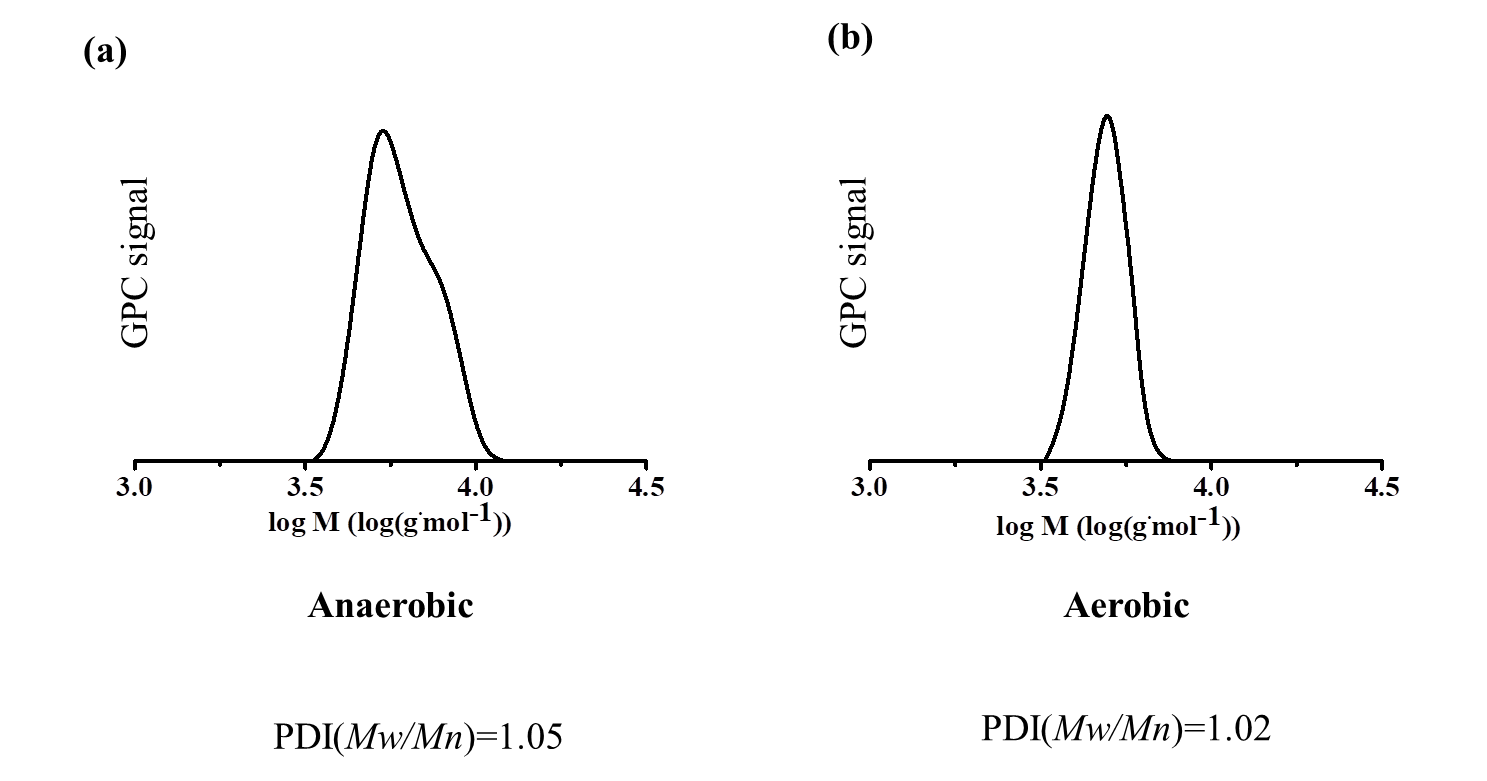


**Fig. S3.** GPC analysis of PSS polymer synthesized by the cytochrome C catalyzed ATRP.


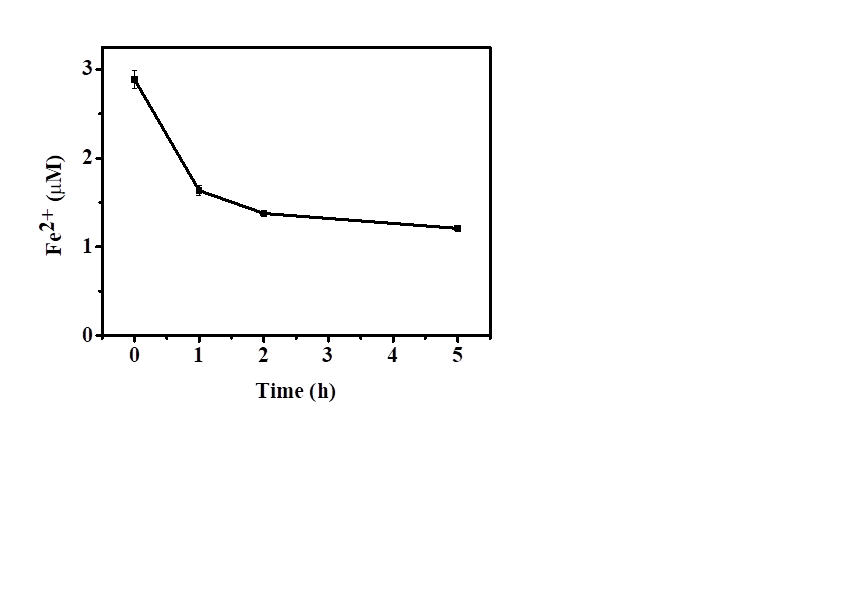


**Fig. S4.** The time-course change of the Fe(II) concentration of the cytochrome C catalyzed ATRP under anaerobic condition.

Experimental details. The o-phenanthroline spectrophotometer method (Berker et al., 2007; Rusevova et al., 2012) was used to determine the concentration of Fe(II). Acetic acid-sodium acetate (136 g of sodium acetate dissolved in 120 mL of acetic acid and diluted with water to 500 mL), HCl:H2O=1:1, hydroxylamine hydrochloride solution (10 g dissolved in 100 mL of pure water), o-phenanthroline solution (0.15 g of o-phenanthroline dissolved in 10 mL of absolute ethanol and diluted with pure water to 100 mL) were prepared. Then, 1 mL of the sample was added into a 10 mL colorimetric tube in anaerobic workstation, 0.1 mL of hydrochloric acid solution was added to the colorimetric tube and wait for 5-10 minutes. Next, add 1 mL acetic acid-sodium acetate solution, 0.5 mL o-phenanthroline into the mixture. Add pure water into the colorimetric tube to 10 mL, shaking thoroughly, and keep it standstill for 10 minutes. Finally, the absorbance was measured at 510 nm and the Fe(II) concentration was calculated based on the calibration curve.


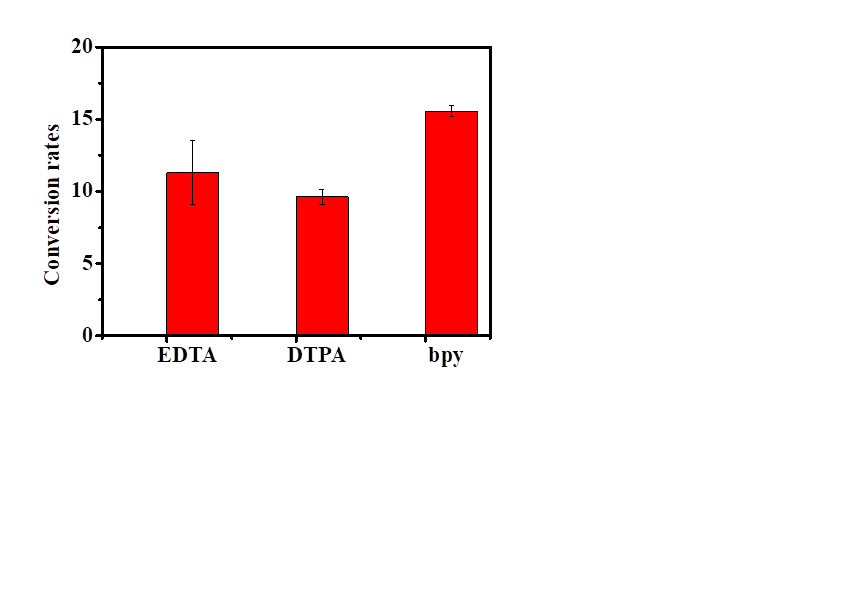


**Fig. S5.** The effects of different metal ion chelators (2 mM) on the SMA conversion of the cytochrome C catalyzed ATRP under anaerobic condition.

Experimental details. Firstly, 5 mL polymerization reaction mixture (cytochrome C (1mM), monomer solution (100 mM SMA), ascorbic acid (10 mM)) was prepared in a 20 mL bottle, and 4 mL PBS were mixed under anaerobic condition. Next, 2mM metal chelator was added, and the initiator BIBB was added. Finally, samples were taken at 5 h of reaction and used for HPLC detection.


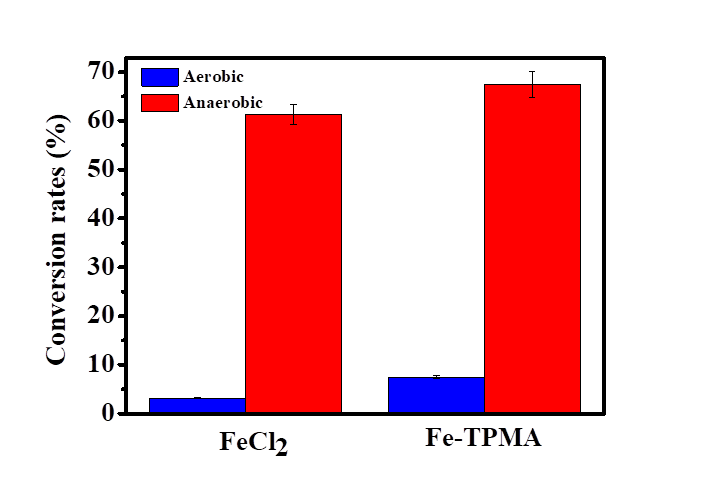


**Fig. S6.** SMA conversion of the free Fe(II) or Fe-TPMA catalyzed ATRP under anaerobic or aerobic condition.


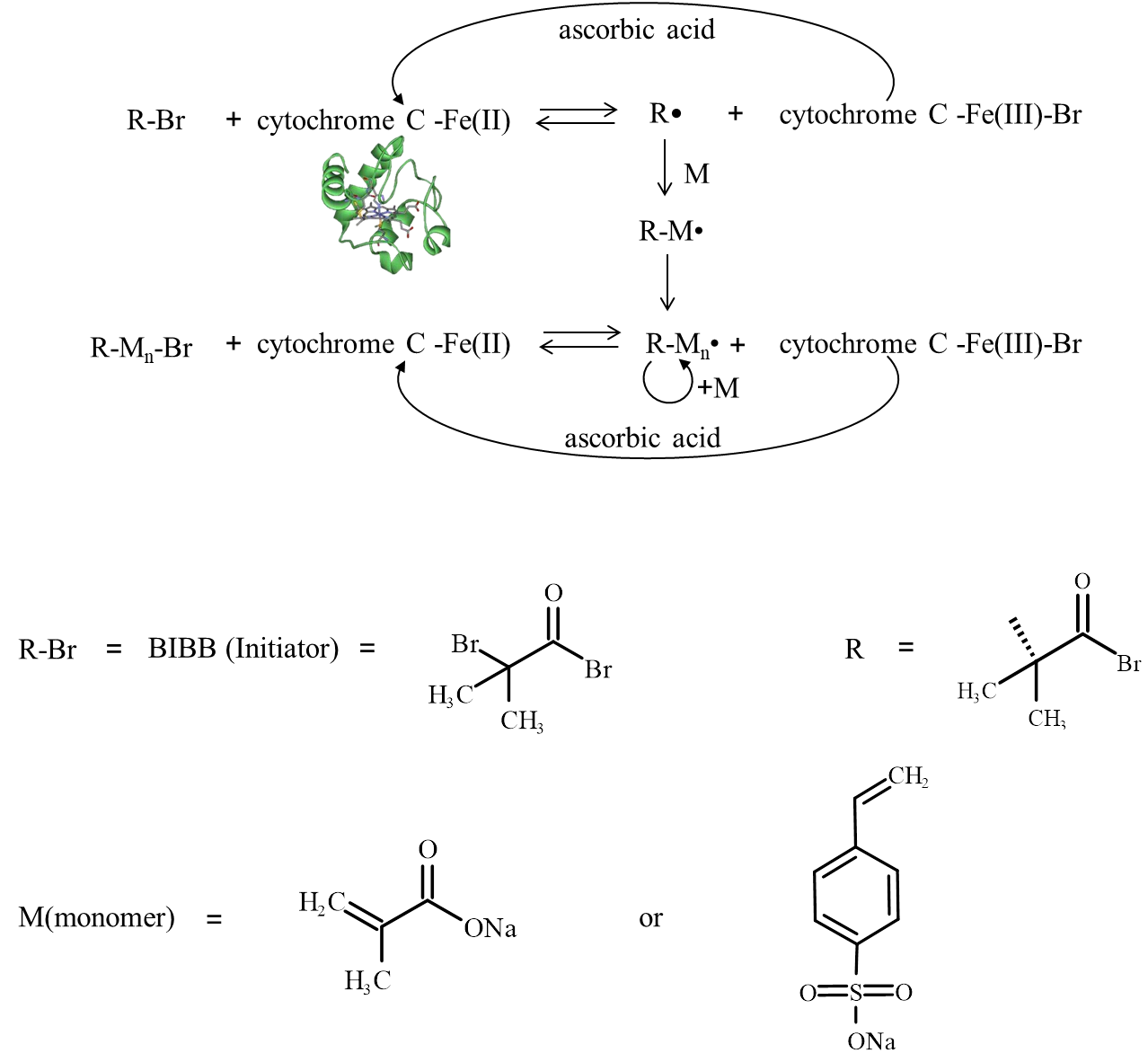


**Fig. S7.** The proposed schematic for cytochrome C catalyzed ATRP.

**References**

Berker, KI, Guclu, K, Tor, I, Apak, R. Comparative evaluation of Fe(III) reducing power-based antioxidant capacity assays in the presence of phenanthroline, batho-phenanthroline, tripyridyltriazine (FRAP), and ferricyanide reagents. Talanta 2007; 72: 1157-65.

Rusevova, K, Kopinke, FD, Georgi, A. Nano-sized magnetic iron oxides as catalysts for heterogeneous Fenton-like reactions-Influence of Fe(II)/Fe(III) ratio on catalytic performance. Journal of Hazardous Materials 2012; 241: 433-440.
